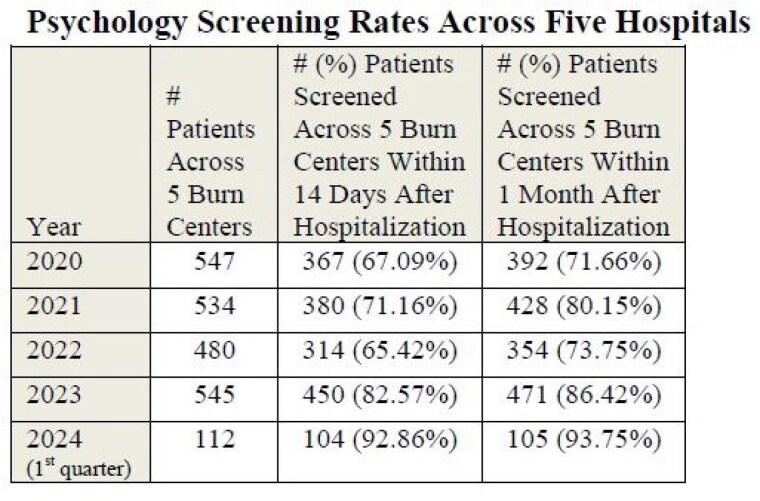# 674 Psychological Screening Rates of Pediatric Patients Across Five Years

**DOI:** 10.1093/jbcr/iraf019.303

**Published:** 2025-04-01

**Authors:** Nicole Caraballo, Carrie Tully, Andrew Gill, Babetta Mathai, Sarah VerLee, Carisa Parrish

**Affiliations:** Children’s Hospital of Michigan Burn Center; Children’s National Hospital; Johns Hopkins University; Nationwide Children’s Hospital; Nationwide Children’s Hospital; Children’s Mercy Kansas City

## Abstract

**Introduction:**

There is a lack of benchmarking available for psychosocial screenings for youth following burn injuries. Five hospitals collaborate in a national quality improvement program as part of an ongoing effort to establish potential benchmarks. We hypothesize that data collection and collaboration across five burn centers will increase psychological screening rates to at least 80% completion, individually and as a group.

**Methods:**

Data were drawn from five centers participating in a national benchmarking quality improvement program June 2019- March 2024. Each program submitted clinical variables (e.g., number of patients admitted for at least 12 hours, psychology screening completed) to a central location. A central data manager kept hospital and patient identity anonymous, and data excluded patient specifiers (e.g., name, age, gender). We examined rates of psychological screening, defined as “clinical psychology screening completed in either inpatient or outpatient setting within 1 month of injury.” We additionally examined rates of psychological screening completed within 14 days of injury.

**Results:**

In the first full year of data available for analysis (2020), 40% of centers met the goal of 80% of patients screened within 1 month of injury, one center met the 80% screening mark for patients within 14 days, and the average screening rate across the five centers was 71.66%. In the last full year of data available for analysis (2023), 80% of centers met the goal of 80% of patients screened within one month of injury, 60% of centers met the 80% screening mark for patients within 14 days, and the average screening rate across centers was 86.42%. For the data available for the first three months of the current year in progress (2024), all centers (100%) reached the 80% screening goal at 1 month and within 14 days of patient injury.

**Conclusions:**

This national survey of psychological screenings completed within pediatric burn centers following a hospitalization indicates variability of screening practice and improvement in screening rates with targeted benchmarks. Collaboration among the five centers to individually and collectively increase screening rates led to identification of multiple variables impacting screening rates (e.g., psychologist FTE dedicated to burn, availability of automatic inpatient psychology consults, structure and frequency of outpatient burn clinics impacting psychologists’ availability for screening).

**Applicability of Research to Practice:**

Defining benchmarks and review of practice data collaboratively across hospitals could assist with improved standardization in care and delivery regarding psychological screenings among pediatric burn patients.

**Funding for the Study:**

N/A